# Artificial intelligence system for predicting hand-foot skin reaction induced by vascular endothelial growth factor receptor inhibitors

**DOI:** 10.1038/s41598-025-93471-x

**Published:** 2025-03-21

**Authors:** Taro Yamanaka, Jumpei Ukita, Dongyi Xue, Chihiro Kondoh, Seiwa Honda, Maiko Noguchi, Yoshiko Yonejima, Kiyomi Nonogaki, Kohji Takemura, Rika Kizawa, Takeshi Yamaguchi, Yuko Tanabe, Koichi Suyama, Keisuke Ogaki, Yuji Miura

**Affiliations:** 1 Department of Medical Oncology, Toranomon Hospital, 2-2-2 Toranomon Minato-ku, Tokyo, 105-8470 Japan; 2 M3 Inc., Tokyo, Japan; 3 Department of Medical Oncology, National Cancer Center Hospital East, Kashiwa, Japan; 4 Mebix Inc., Tokyo, Japan

**Keywords:** HFSR, VEGFR inhibitor, Deep learning, AI, Cancer, Cancer therapy, Skin manifestations, Computer science

## Abstract

Hand-foot skin reaction (HFSR) is a common adverse effect of vascular endothelial growth factor receptor (VEGFR) inhibitors that significantly impacts patients’ quality of life. Prevention and management of HFSR require individualized approaches, but risk factors remain unclear. This study aimed to develop artificial intelligence (AI) models to predict grade ≥ 2 HFSR using clinical data and foot sole images from 93 instances of VEGFR inhibitor administration in 76 patients. Image-based, clinical information-based, and ensemble AI models achieved areas under the curve of 0.550, 0.693, and 0.699, respectively. At a high-specificity cutoff, the ensemble AI had a positive predictive value of 0.824, suggesting potential clinical utility for identifying high-risk patients. Feature importance analysis revealed heavier weight, good performance status, lack of prior VEGFR inhibitor exposure, and baseline skin toxicity as risk factors. These findings represent the first AI-based HFSR prediction models and provide insights for preventive interventions, but further accuracy improvements are needed.

**Supplementary Information:**

The online version contains supplementary material available at 10.1038/s41598-025-93471-x.

## Introduction

Hand-foot skin reaction (HFSR) is a condition primarily affecting the skin on the palms of the hands and soles of the feet. It is caused by multi-kinase inhibitors, such as vascular endothelial growth factor receptor (VEGFR) inhibitors. HFSR is classified as palmar-plantar erythrodysesthesia according to the National Cancer Institute’s Common Terminology Criteria for Adverse Events (NCI-CTCAE), but it is distinct from hand-foot syndrome, which is associated with chemotherapeutic agents such as 5-fluorouracil and capecitabine. Clinical manifestations of HFSR include skin redness, swelling, and a tingling sensation in mild cases; however, in more severe cases, skin peeling, blisters, and edema with pain can significantly impact the patients’ quality of life. The incidence of HFSR induced by VEGFR inhibitors varies from drug to drug and it is reported to be 20–47% for all grades and 5–17% for grade 3 or higher^1–5^. In addition, the incidence of HFSR is higher in Asian populations than in other groups^6–8^.

Prevention and treatment of HFSR are largely based on expert opinions because of the paucity of evidence. Several prophylactic measures are recommended, including (1) removing or softening preexisting hyperkeratotic areas or calluses using urea cream, (2) using moisturizing cream, (3) reducing pressure on the feet while wearing thick cotton socks or shoes with padded insoles, (4) avoiding constrictive footwear to avoid excessive friction, and (5) avoiding excessive exposure to hot water through dishwashing or hot baths and showers^9^. The effective implementation of these preventive measures requires educating each patient and implementing sustained management by experienced healthcare providers who can assess the risk based on baseline skin conditions and tailored interventions to the individual lifestyle of each patient.

Artificial intelligence (AI) systems are evolving and are being applied clinically in various fields of medicine. Currently, various AI systems provide detailed diagnoses with high accuracy based on existing pathological and radiological information^10,11^. However, AI systems for predicting the prognosis of patients have not been extensively investigated. Few studies have predicted the future occurrence of drug side effects using easily accessible patient data^12–14^. Here, we aimed to develop an AI system to predict the future occurrence of HFSR based on the patient’s clinical information and photographs of the foot soles before the administration of VEGFR inhibitors, which may be helpful even for inexperienced healthcare providers to provide an effective personalized approach to HFSR. To our knowledge, this is the first study applying AI for HFSR risk stratification.

## Results

### Patient characteristics

The analyzed cohort comprised 93 VEGFR inhibitor administrations among 76 patients. The median age was 63 years (range, 39–83), and 64.5% of the patients were male. Performance statuses of 0, 1, 2, unknown were 46 (49.5%), 29 (31.2%), 5 (5.4%), and 13 (14.0%), respectively. The most common VEGFR inhibitor used in this cohort was regorafenib (*n* = 39 [41.9%]), followed by axitinib (*n* = 20 [21.5%]), sunitinib (*n* = 11 [11.8%]), pazopanib (n = 11 [11.8%]), and others (*n* = 12 [12.9%]). The most common cancer type in this cohort was renal cell carcinoma (n = 41 [44.1%]), followed by colorectal cancer (*n* = 35 [37.6%]) and gastrointestinal stromal tumors (*n* = 12 [12.9%]). Thirty-five patients had Grade 1 skin toxicity in their sole at baseline. The cause of skin toxicity was HFSR induced by previous administration of VEGFR inhibitor in 15 patients (42.9%); and hand-foot syndrome induced by previous fluoropyrimidine, anti-epidermal growth factor receptor (EGFR) antibody, and trifluridine-tipiracil in 16 patients (45.7%); and others in 4 patients. The detailed patient characteristics are shown in Table 1.

**Table 1:** Patient characteristics.

	*N* (%)
Sex	
Male	60 (64.5)
Female	33 (35.5)
Age	
Median (range)	63 (39–83)
Performance status	
0	46 (49.5)
1	29 (31.2)
2	5 (5.4)
Unknown	13 (13.9)
Cancer type	
Renal cell carcinoma	41 (44.1)
Colorectal	35 (37.6)
GIST	12 (12.9)
Thyroid	3 (3.2)
Soft tissue sarcoma	2 (2.2)
Type of VEGFR inhibitor	
Regorafenib	39 (41.9)
Axitinib	20 (21.5)
Sunitinib	11 (11.8)
Pazopanib	11 (11.8)
Cabozantinib	7 (7.5)
Lenvatinib	3 (3.2)
Sorafenib	2 (2.2)

GIST: gastrointestinal stromal tumor, VEGFR: vascular endothelial growth factor receptor.

### Overall incidence of HFSR

The overall incidence of any grade HFSR was 77.4% (grade 1: 28.0%; grade 2: 31.2%; grade 3: 18.3%).

### Receiver operating characteristic curve, sensitivity, and specificity

**Fig. 1:**
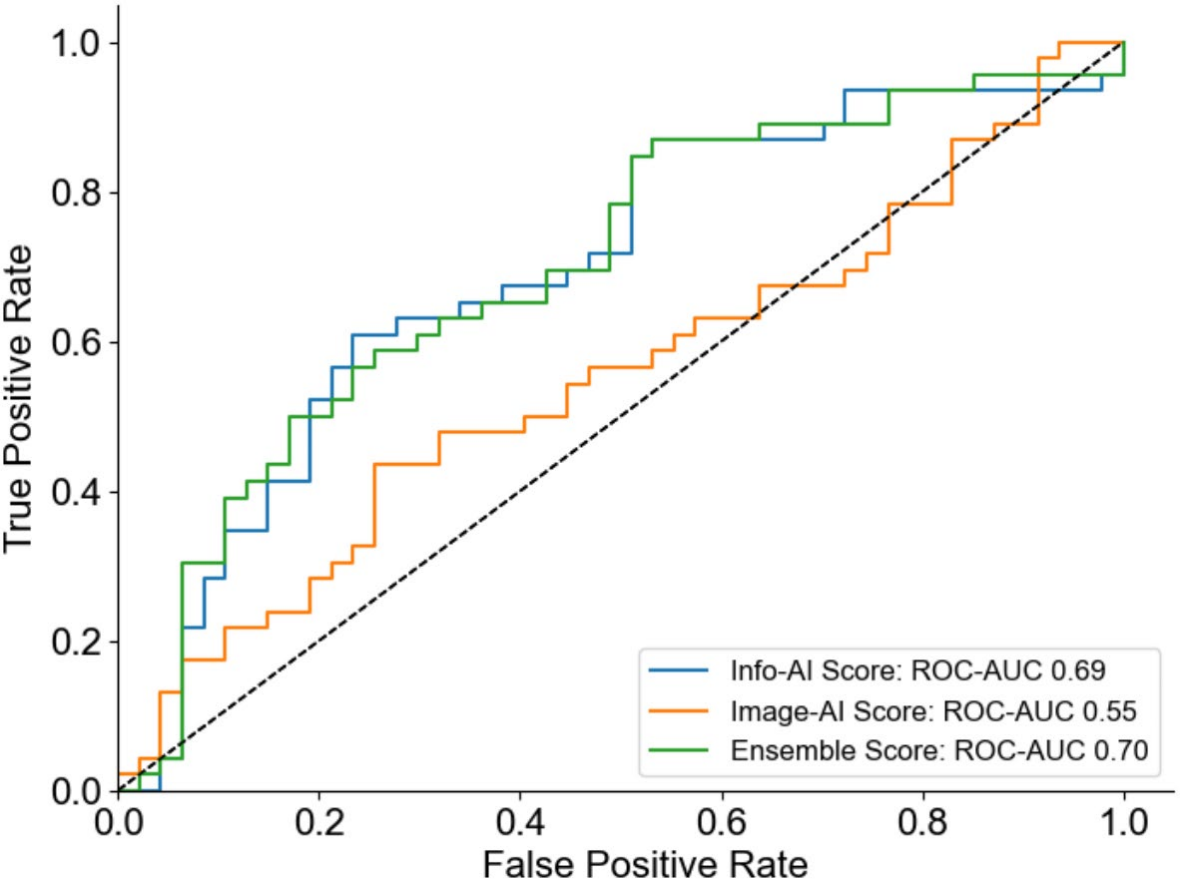
Receiver operating characteristic curve. The Image-AI, Info-AI and Ensemble-AI models demonstrated orange, blue and green curves, respectively. ROC-AUC, Receiver operating characteristic curve-area under the curve.

Figure 1 shows the receiver operating characteristic (ROC) curve for the overall population. The image-based (Image-AI), clinical information-based (Info-AI), and ensemble-AI models exhibited areas under the curve (AUC) of 0.550, 0.693, and 0.699, respectively, for predicting grade ≥ 2 HFSR. Table 2 shows the results of the stratified analysis, which included the subgroups of (1) patients with or without skin toxicity at baseline and (2) patients with or without previous administration of VEGFR inhibitors. Little difference in the AUC values from Image-AI was observed in each subgroup; however, the AUC values from Info-AI and Ensemble-AI were higher in the subgroup without baseline hand-foot syndrome or a previous VEGFR inhibitor. At a high-specificity cutoff, the sensitivity, specificity, positive predictive value, and negative predictive value of the ensemble-AI were 0.304, 0.936, 0.824, and 0.579, respectively (Table 3).

**Table 2:** Stratified analysis of AUC.

AUC	Info-AI	Image-AI	Ensemble-AI
Skin toxicity at baseline			
Grade 0 (*n* = 58)	0.77	0.505	0.762
Grade 1 (*n* = 35)	0.528	0.559	0.542
Previous VEGFR inhibitor			
No (*n* = 63)	0.688	0.527	0.695
Yes (*n* = 30)	0.497	0.508	0.540

AUC: area under the curve, VEGFR: vascular endothelial growth factor receptor, AI: artificial intelligence.

**Table 3:** Sensitivity, specificity, positive predictive value, and negative predictive value of Ensemble-AI with different cutoff values.

	Sensitivity	Specificity	PPV	NPV
High sensitivity cutoff	0.848	0.489	0.619	0.767
High specificity cutoff	0.304	0.936	0.824	0.579

PPV: positive predictive value, NPV: negative predictive value.

### Risk factors from baseline clinical information

**Fig. 2:**
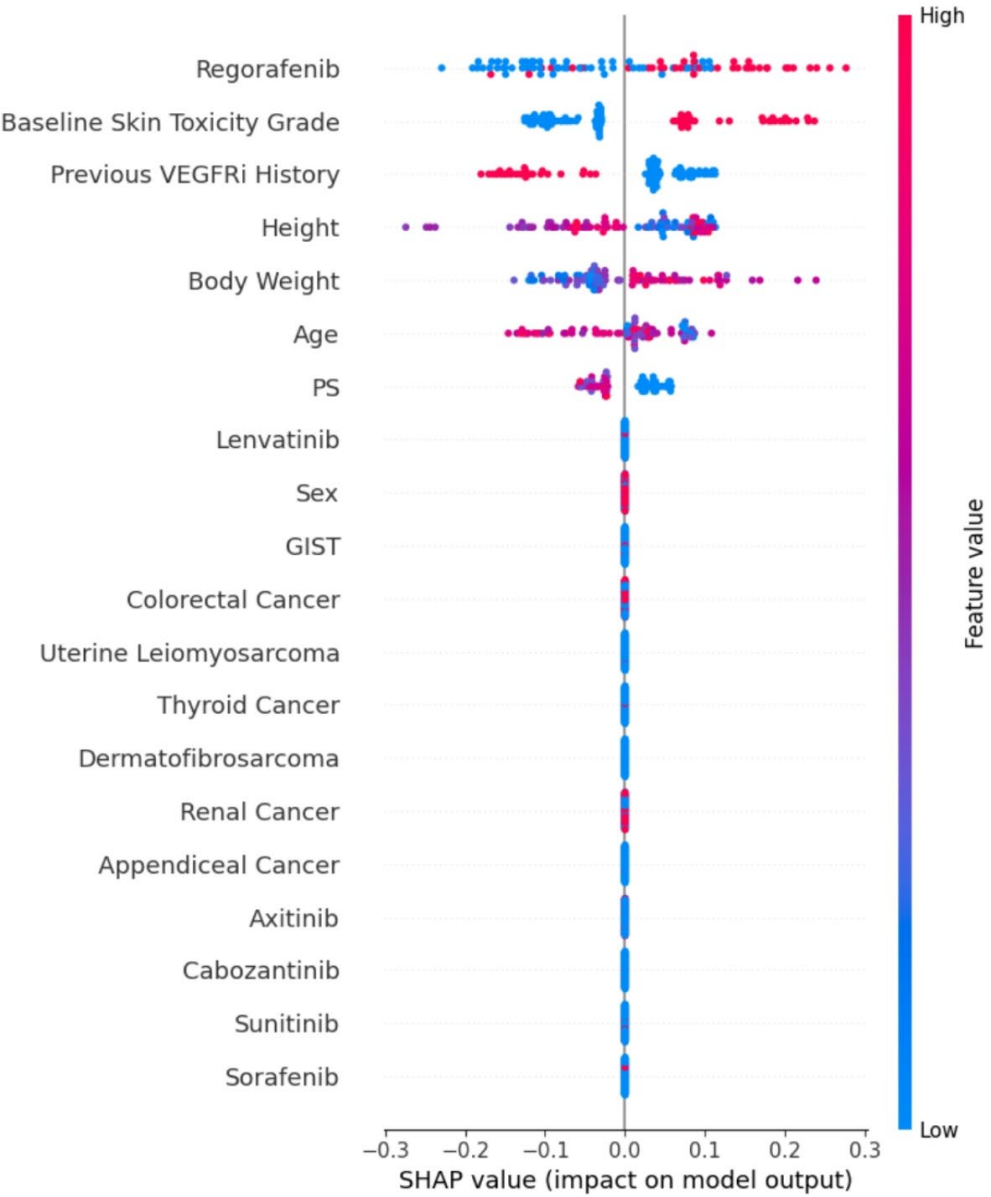
SHAP values. Positive SHAP values indicate a positive impact on the development of HFSR. The color bars demonstrate that the higher and lower values for each factor are shown in red and blue, respectively. VEGFRi, vascular endothelial growth factor receptor inhibitor; PS, performance status; GIST, gastrointestinal stromal tumor.

To understand the importance of clinical factors in the info-AI model, we calculated SHapley Additive exPlanations (SHAP) values (Fig. 2). Regorafenib use, baseline skin toxicity, no prior use of VEGFR inhibitors, heavier weight, and good performance status were considered the top predictors of HFSR development.

## Discussion

We developed an AI system to predict the future onset of HFSR based on plantar images and clinical information prior to VEGFR inhibitor administration. The ensemble model integrating clinical information and foot images showed modest performance (AUC 0.699), while at a high-specificity cutoff, the AI system showed a certain PPV (0.824) and may be clinically applicable for predicting high-risk patients for developing HFSR. Intensive care for high-risk patients may reduce the occurrence of HFSR. However, the low sensitivity (0.304) indicates many cases were failed to predict HFSR, highlighting the need for further improvements.

As the research and development of medical AI systems for predicting future conditions continue, various problems remain to be solved. First, the information necessary to predict HFSR may not have been included in the model input. For example, prophylactic measures provided during treatment were not incorporated into the model input, although they may be useful features for the prediction task. Therefore, the task itself may be inherently difficult even for medical experts. Second, the amount of data used to develop the models may not have been sufficient (76 cases and 93 drug administrations). To verify this possibility, we analyzed the relationship between the sample size and the accuracy of the AI system. When X% of the amount of data collected in this study was sampled and used to train and evaluate the AI (X = 50, 55, …, 100), the accuracy increased monotonically with X for all the Image-AI, Info-AI, and Ensemble-AI models (Supplementary Fig. 1A, B, and C). These results suggest that the accuracy can be further improved by collecting more data than those collected in this study. This tendency was particularly strong for the Image-AI model, suggesting that increasing the amount of data could be especially helpful in improving the accuracy of the Image-AI model. Third, the quality of the images used for Image-AI may need to be improved. Excessive skin rubbing and pressure are known to cause HFSR^9^; therefore, not only the sole but also the lateral side of the foot is a common location for its development. However, the current study used photographs of the sole in only one direction, which may have failed to predict the HFSR on the lateral side of the foot, thereby reducing the overall accuracy of the study. This problem may be resolved by utilizing multidirectional or three-dimensional photography. Fourth, the retrospective design precluded standardized data collection on potentially relevant variables that could have affected the outcomes of HFSR development, such as the use of moisturizing creams, urea creams, and topical corticosteroids and dose reduction or interruption of VEGFR inhibitors. Further prospective studies are warranted. Fifth, while we employed cross-validation, a widely accepted method for evaluating machine learning models^14^, our study did not include validation using an external test set. Additionally, our dataset was collected from a single institution in Japan, introducing potential population bias. These limitations may raise two concerns regarding generalizability of our model: the replicability across different institution within Japan, and the applicability to populations of different ethnic backgrounds, given known variation in HFSR incidence across ethnicities. Future multi-institutional studies incorporating diverse patient population will be essential to validate and expand the applicability of our findings.

In the present study, the accuracy of Image-AI was lower than that of Info-AI. Image AI is based on a deep learning model that has more parameters and generally requires more data for training than non-deep learning models. Together with the results shown in Supplementary Fig. 1, the difference in accuracy between Info-AI and Image-AI may be partially explained by the amount of data required to train the models.

Interestingly, in this study, relatively high AUC values were obtained using the Info-AI model. Notably, SHAP analysis revealed that patients with a heavier weight and a performance status of 0 rather than 1 had a greater risk of developing HFSR, which aligns with reports linking the occurrence of HFSR to pressure-bearing locations and skin friction^9^. Furthermore, guidance on pressure relief methods, such as using a soft insole, avoiding stiff-soled shoes or tight shoes, and lifestyle modifications, such as avoiding excessive walking, may be suggested as effective preventive measures. Additionally, the patients with baseline skin toxicity and those without previous use of VEGFR inhibitors were at high risk of developing HFSR. Among the 35 patients with baseline skin toxicity, 16 and 14 patients had prior exposure to fluorouracil and VEGFR inhibitors, respectively, suggesting that the patients with fluorouracil-induced skin toxicity might represent a particularly susceptible population. Moreover, SHAP analysis revealed that patients treated with regorafenib were at higher risk for HFSR development. This result may be explained by the known tendency of regorafenib to cause high-grade HFSR more frequently compared to other VEGFR inhibitors, as reported in previous study^15^. Furthermore, when used for colorectal cancer, the increased risk might be attributable to prior long-term exposure of fluorouracil, which is typically part of the standard treatment regimen in these patients. However, due to the limited number of such patients in our study, statistical validation of these findings was not feasible. Further investigation with large patient population is warranted.

In conclusion, we present the first AI-based HFSR prediction models. While promising for risk stratification, further development is needed to improve accuracy for clinical deployment. Larger, prospective studies with standardized multimodal data collection may help refine these models to enhance personalized HFSR prevention and management.

## Methods

### Data collection

We retrospectively analyzed the database of the Oral Chemotherapy Support Team and medical records of patients who received VEGFR inhibitors at Toranomon Hospital between January 2014 and June 2021. The following information was collected: clinical background at the start of VEGFR inhibitor treatment, including age, sex, height, body weight, Eastern Cooperative Oncology Group (ECOG) performance status, cancer type, baseline skin condition, type of VEGFR inhibitor, and photographs of the sole of the foot within one week before starting VEGFR inhibitors. The documented CTCAE grade of HFSR was retrospectively collected from the electronic medical records, and in cases without evaluation of the grading in electronic medical records, we assessed the grading based on photographs and the patient’s complaints or condition, as documented in the medical records.

Eighty patients (equivalent to 97 drug administrations because the same patient received several different types of VEGFR inhibitors in 17 patients with adequate photographs of the sole for AI) were extracted from the database. Four patients were excluded from the current study because they had grade 2 or higher HFSR or hand-foot syndrome in their sole induced by previous administration of chemotherapeutic agents at the time of VEGFR inhibitor administration. Additionally, if a patient used multiple VEGFR inhibitors sequentially, we considered them separately; consequently, in the present study, 76 patients and 93 drug administrations were included in the final analysis. We developed and evaluated the AI system using 93 separate cases of drug administration.

### Ethics declarations

The Institutional Review Board of Toranomon Hospital approved the study (approval number: 2041) in accordance with the principles of the Declaration of Helsinki of 1964 and its later versions.

### Consent to participate

The requirement for informed consent from patients was waived by the Institutional Review Board of Toranomon Hospital through the use of an opt-out method. The purpose and methods of the research were posted on the website of Toranomon Hospital, ensuring that patients had the opportunity to refuse participation in the study.

### Machine learning methods

Two separate models were created: (1) a model for predicting the occurrence of grade 2 or higher HFSR from foot images (Image-AI) and (2) a model for predicting the occurrence of grade 2 or higher HFSR from clinical information (Info-AI). After developing these two models, (3) a model that ensembles the outputs of (1) and (2) (Ensemble-AI) was created, and the output of (3) was used as the final output. The reason for using this type of late fusion for the ensemble of image and non-image information was to easily compare the accuracy when (1) images only, (2) clinical information only, or (3) both were used as the model inputs.

During the development of Image-AI, the images were preprocessed using Otsu’s binarization method to remove the background. The images were augmented by random color jitter (brightness factor range, [− 0.2, 0.2]; contrast factor range, [− 0.2, 0.2]; and probability, 0.5), random vertical flip (probability: 0.5), random horizontal flip (probability: 0.5), random rotation (angle range: [− 7, 7], probability: 0.5), and random gamma correction (gamma range: [80, 120], and probability: 0.5). During training and testing, the images were processed such that the pixel intensities were between 0 and 1 and the resolution was 256 × 256. A single model consisting of ResNet50^16^ pretrained on the ImageNet^17^ database was used as the feature extractor, and a fully connected layer was concatenated on top of the feature extractor. A single model was trained for 30 epochs to minimize the binary cross-entropy loss using the Adam optimizer with the following hyperparameters: batch size of eight and learning rate of 0.0001. We developed and evaluated the models using patient-wise 4-fold stratified cross validation with five different random seeds and averaged the outputs across seeds to produce the output of Image-AI.

When developing the Info-AI, XGBoost with the following hyperparameters was used as a single model: the minimum child weight was three and the maximum depth was five. The missing values were imputed using the average of the features. Similar to Image-AI, we developed and evaluated the models using patient-wise 4-fold stratified cross validation with 10 different random seeds and averaged the outputs across seeds to produce the output of Info-AI. The Ensemble-AI was averaged over the outputs of Image-AI and Info-AI.

When investigating the SHapley Additive ExPlanations (SHAP) values^18^ for Info-AI, the model was retrained using all datasets.

Development and evaluation were performed in Python 3.7 using PyTorch 1.6.0, Torchvision 0.7.0, Albumentations 1.3.0, and xgboost 1.6.2.

## Electronic supplementary material

Below is the link to the electronic supplementary material.

Supplementary Material 1

Supplementary Material 2

## Data Availability

The data that support the findings of this study are available on request from the corresponding author, [YM]. The data are not publicly available because they contain containing information that could compromise research participant privacy.
