# Supplementary figures and images for: Artificial intelligence system for predicting hand-foot skin reaction induced by vascular endothelial growth factor receptor inhibitors
Source: Sci Rep. 2025 Mar 21;15:9843. doi: 10.1038/s41598-025-93471-x (PMC11928579; doi:10.1038/s41598-025-93471-x)

## Slide 1
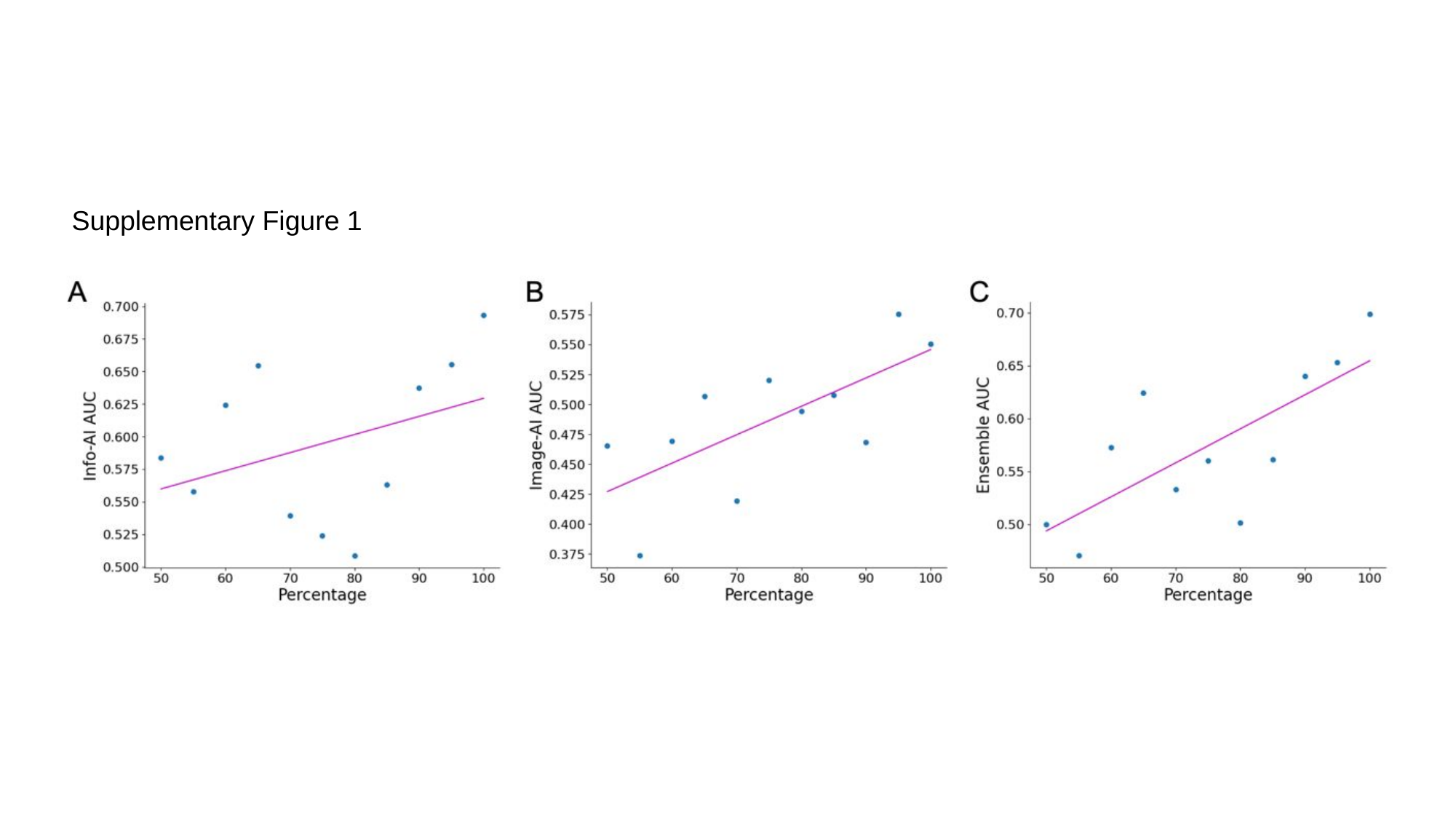

Supplementary Figure 1

Supplement: Supplementary file 1 — Supplementary Material 1 [file 41598_2025_93471_MOESM1_ESM.pptx]
