# Supplementary material for: Artificial intelligence system for predicting hand-foot skin reaction induced by vascular endothelial growth factor receptor inhibitors
Source: Sci Rep. 2025 Mar 21;15:9843. doi: 10.1038/s41598-025-93471-x (PMC11928579; doi:10.1038/s41598-025-93471-x)
Supplement: Supplementary file 2 — Supplementary Material 2 [file 41598_2025_93471_MOESM2_ESM.docx]

Supplementary Figure 1. The relationship between sample size and accuracy of the AI system.

The relationship between sample size and the AUC of A) Info-AI, B) Image-AI and C) ensemble -AI. AUC: area under the curve, AI: artificial intelligence.
